# Calcium Dynamics in Root Cells of *Arabidopsis thaliana* Visualized with Selective Plane Illumination Microscopy

**DOI:** 10.1371/journal.pone.0075646

**Published:** 2013-10-16

**Authors:** Alex Costa, Alessia Candeo, Luca Fieramonti, Gianluca Valentini, Andrea Bassi

**Affiliations:** 1 Dipartimento di Bioscienze, Università degli studi di Milano, Milano, Italy; 2 Dipartimento di Fisica, Politecnico di Milano, Milano, Italy; University of Nottingham, United Kingdom

## Abstract

Selective Plane Illumination Microscopy (SPIM) is an imaging technique particularly suited for long term *in-vivo* analysis of transparent specimens, able to visualize small organs or entire organisms, at cellular and eventually even subcellular resolution. Here we report the application of SPIM in Calcium imaging based on Förster Resonance Energy Transfer (FRET). Transgenic Arabidopsis plants expressing the genetically encoded-FRET-based Ca^2+^ probe Cameleon, in the cytosol or nucleus, were used to demonstrate that SPIM enables ratiometric fluorescence imaging at high spatial and temporal resolution, both at tissue and single cell level. The SPIM-FRET technique enabled us to follow nuclear and cytosolic Ca^2+^ dynamics in Arabidopsis root tip cells, deep inside the organ, in response to different stimuli. A relevant physiological phenomenon, namely Ca^2+^ signal percolation, predicted in previous studies, has been directly visualized.

## Introduction

Calcium (Ca^2+^) is a multifaceted second messenger in eukaryotic organisms. In plants, Ca^2+^ is involved in many aspects of development and takes part into different regulatory processes [1], [2]. Plant cells respond to several environmental or developmental stimuli, by changing the intracellular free Ca^2+^ concentration. These changes are commonly referred as “Ca^2+^ signatures” and can range from a single transient increase to a series of repetitive Ca^2+^ oscillations [3]–[7]. The leading hypothesis states that different Ca^2+^ signatures might encode specific information, leading to distinct downstream responses [3]–[9]. Several examples in support of such hypothesis have been reported in single cell systems, such as guard cells [3], [4] and root hairs of leguminous species [10] but also in entire seedlings. In the latter case, imposing distinct Ca^2+^ elevations differentially affected gene expression [11], [12]. The introduction of genetically encoded Ca^2+^ indicators (e.g. aequorin and GFP-based Ca^2+^ probes), has permitted the detection and visualization of intracellular Ca^2+^ dynamics in living plants [13], [14]. Experiments performed using aequorin produced reliable data, but could only show the response of a population of cells or plants, not allowing the investigation of intercellular heterogeneities. In order to improve cellular and subcellular resolution, the use of other Ca^2+^ sensors has been pursued in recent years and, among the genetically encoded Ca^2+^ indicators, Cameleons are the most frequently used [14]–[20]. Cameleons are Förster Resonance Energy Transfer (FRET) based indicators in which two fluorescent proteins, CFP and YFP (or circularly permuted variants of YFP), are linked together by the calcium-binding protein calmodulin and a calmodulin binding peptide. Binding of Ca^2+^ to these calcium-responsive elements alters the distance between the two fluorophores hence the efficiency of FRET, allowing a quantitative measurement of Ca^2+^ dynamics [21]. In particular, one of the most employed FRET probes in plant biology is the Yellow Cameleon YC3.6 [22], which has been specifically designed to improve the brightness and energy transfer between the FRET couple, CFP and cpVenus. The YC3.6 probe has several peculiar properties that fit well the needs of plant biology: i) high signal to noise ratio; ii) high dynamic range; iii) pH stability in the physiological range; iv) an *in vitro* Kd for Ca^2+^ of 250 nM [22]. All these features make Cameleon YC3.6 suitable for Ca^2+^ sensing in different cell types and possibly in subcellular compartments [19], [20], [23].

The *in-vivo* visualization of FRET sensors in complex plant organs/tissues such as the root, is possible with standard microscopy modalities, all of which present different limitations.

Wide-field microscopy has been successfully used with the main limitation of absence of depth sectioning [19], [20], the Ca^2+^ response is averaged over the entire volume, analysis at single cell resolution is not possible and, as a result, only the averaged cellular response can be observed. Single cell resolution in the root has been achieved by means of confocal laser scanning microscopy (CLSM) using Cameleon probes in response to both biotic and abiotic stimuli [16], [17], [19], [24]. However, CLSM can cause strong photo-bleaching (in particular for repetitive three-dimensional measurements), the acquisition is slow or limited to a small volume, restricting the analysis to single or few cell layers [17], [19], [24], [25]. Furthermore, in the majority of microscopes the plant is mounted horizontally on a glass slide. Although largely used, such specimen preparation can itself induce stress conditions or physiological responses such as hormone/s redistribution. A simple case occurs in the root tissues, where the auxin distribution is known to be regulated in response to a gravitropic stimulus [26].

**Figure 1:**
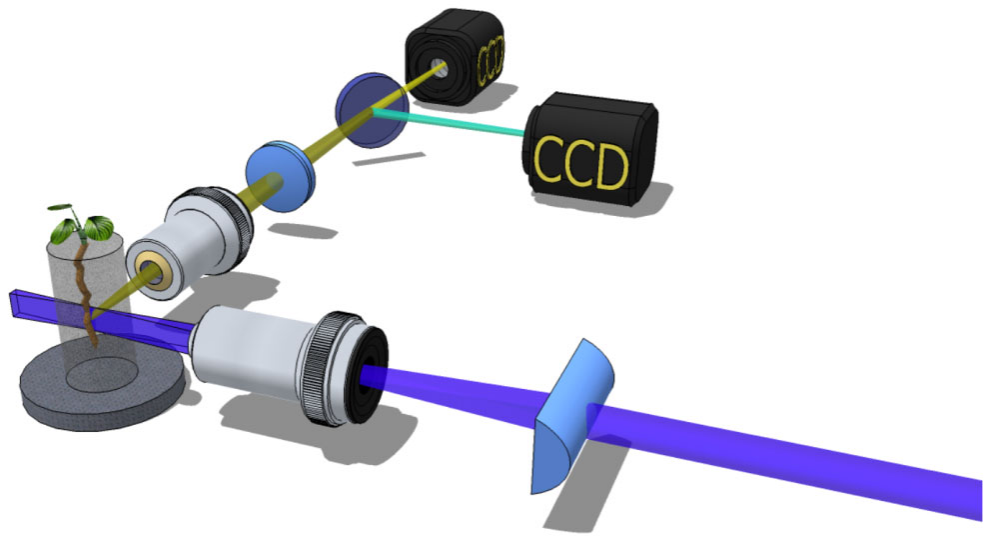
Schematic of the SPIM-FRET setup: a cylindrical lens in combination with a microscope objective create a light-sheet on the sample orthogonal to the detection axis. Two CCDs are used to image the CFP (Fluorescence) and cpVenus (FRET) signals. Note that for the simultaneous acquisition of the two channels, several configurations are possible: i) two independent cameras; ii) a camera with two CCDs; iii) a single camera coupled with a beam splitter can be used (not shown in the cartoon).

In order to overcome these limitations, we used Selective Plane Illumination Microscopy (SPIM) to image Arabidopsis transgenic plants expressing the nuclear and cytosolic-targeted Cameleon YC3.6, previously generated by Schumacher and coworkers [19]. The system is equipped with parallel detection of two wavelength channels for ratiometric FRET imaging (Figure 1). SPIM [27] is a fluorescence microscopy technique, in which the illumination axis is perpendicular to the detection axis. A laser beam is used to create a thin light-sheet right in the focal plane of the detection objective. The light-sheet can be a few microns thin thus avoiding out of focus fluorescence and providing optical sectioning. The lateral resolution is given by the detection objective, yielding subcellular details. SPIM is well suited for rapid 3D *in-vivo* imaging of *Arabidopsis thaliana* specimens at high spatial resolution, with minimal photo-damage to the sample [28], [29]. In fact, only the plane of the specimen under observation is illuminated. This configuration greatly reduces the light dose and therefore photo-bleaching and photo-toxicity. Another advantage of SPIM is that, during the entire analyses, the seedlings are placed in conditions similar to those used for their growth, with the root floating and growing in the water based solution and the shoot in free air (Figure 1).

## Materials and Methods

### SPIM apparatus

The microscope is a modified version of the OpenSPIM project [30], and it is similar to a single illumination arm SPIM [31]. A single mode fiber coupled laser at 442 nm (MDL-III-442, CNI) is collimated and used for SPIM illumination. An automatic shutter switches the beam on and off via computer control. A cylindrical lens focuses the light in a horizontal plane and a 1× telescope images the focal plane of the cylindrical lens in the back focal plane of the illumination 10× water dipping microscope objective (UMPLFLN 10XW, Olympus). As a result, a vertical light-sheet is created on the sample in the front focal plane of this objective. A slit placed in the center of the telescope confines the excitation light-sheet within the imaged area. The height of the light-sheet is 600 µm and its thickness is about 3 µm (beam waist in the focal plane of the illumination objective). Typical illumination power at the sample was between 10 and 50 µW. We didn't observe any photo-bleaching (during continuous illumination for more than 5 minutes) for illumination powers below 15 µW. The detection unit consists in a 20× water dipping microscope objective (UMPLFLN 20XW, Olympus), a tube lens (U-TLU-1-2, Olympus), and a dual sensor CCD camera (Orca D2, Hamamatsu Photonics K.K.). The illumination and the detection microscope objectives are precisely aligned at 90° in the imaging chamber, which is filled with a water-based solution. The detector consists of a dual-CCD system in which the two sensors are positioned at 90° after a dichroic filter (at 510 nm). One of the two sensors can be rotated and translated to correct focus and alignment in order to produce high contrast images. In addition to the dichroic filter, two band-pass filters (centered at 483 and 542 nm) are used to detect CFP and cpVenus (FRET) signals simultaneously. A white-LED illuminator in transmitted light configuration is used during the alignment of the sample to minimize the exposure to the laser light.

### Sample preparation

The sample consists of 12–14 day-old transgenic Arabidopsis seedlings expressing the cytosolic (NES-YC3.6) or nuclear (NLS-YC3.6) localized Cameleon YC3.6. The generation of these lines was previously reported in Ref. [19] in which the full description of the targeting strategies is reported. The seeds are surface sterilized by vapor-phase sterilization and directly placed, with a toothpick, over a conical plastic holder (typically a 10 µL pipette tip) [32]. The holder is filled with half strength Murashige and Skoog medium (MS, M0222 elements including Vitamins, Duchefa, http://www.duchefa-biochemie.nl/) [33] supplemented with 0.1% sucrose, 2.34 mM MES with a final pH of the media to 6.0±0.1 with 0.5 M KOH and 0.8% of micro agar (Duchefa). The plastic holders are then transferred to a transparent plastic box filled with sterile half strength MS solution for hydroponic culture and placed in a growth chamber under 16/8 h cycles of white light at 22°C. The hydroponic system allows the seedling roots to grow, following the positive gravitropism, first into the agar and subsequently directly in hydroponic solution when they reach the bottom hole of the holder. Once the root comes out the hole of the plastic holder, the specimen is transferred to the SPIM-FRET setup into the imaging chamber filled with the desired solution (for our Ca^2+^ dynamic analyses a 10 mM MES, 5 mM KCl, 10 mM CaCl_2_, pH 5.8 adjusted with TRIS-BASE solution was employed). This procedure prevents any kind of damage or major stress to the root and maintains the seedling vertical. For the analysis of spatiotemporal dynamics of the [Ca^2+^] rise, a volume of 120 µL (100X) glutamate (L-Glu) or external ATP (eATP) was directly added to one corner of the imaging chamber (filled with 12 mL of imaging solution). The final concentration of the stimuli was 1 mM and 0.1 mM for L-Glu and eATP respectively. The stock ATP solution was diluted in a TRIS buffer (pH 5.8) in order to prevent any pH change of the imaging solution.

### Imaging procedure

In the course of SPIM acquisition the root was imaged simultaneously by the 2 CCDs of the dual-sensor camera (Orca D2, Hamamatsu Photonics K.K.) and automatically translated with steps of 1 to 10 µm to create a 3D reconstruction of a large tissue volume by generating multiple image stacks. The shutter, the translation stage and the camera were controlled using Micro-Manager (www.micro-manager.org). This software enables the user to easily program the scanning of the sample over the light sheet and time-lapse acquisition parameters at the beginning of each experiment. The exposure time for each plane was typically 50–500 ms. Two different types of acquisitions were carried out: i) two-dimensional analysis, in which a single plane was imaged with continuous illumination and multiple acquisitions lasting 60–300 s; ii) three-dimensional analysis, in which several planes (typically 10–20) were acquired successively: every 5–10 s the acquisition of the entire stack was repeated. The fluorescence intensity was determined over Regions Of Interests (ROIs) corresponding to large root tip areas, single cells or nuclei. Background subtraction was performed in each channel before FRET ratio calculation by selecting a ROI outside the sample. The FRET ratio (R) was calculated and visualized using the Ratio Plus Plugin for Fiji (http://fiji.sc/) or NisElement (Nikon). For time course experiments the change in the FRET ratio (ΔR) was normalized to the initial value (R_0_) and plotted *versus* time (ΔR/R_0_).

## Results and Discussion

### Image quality

**Figure 2:**
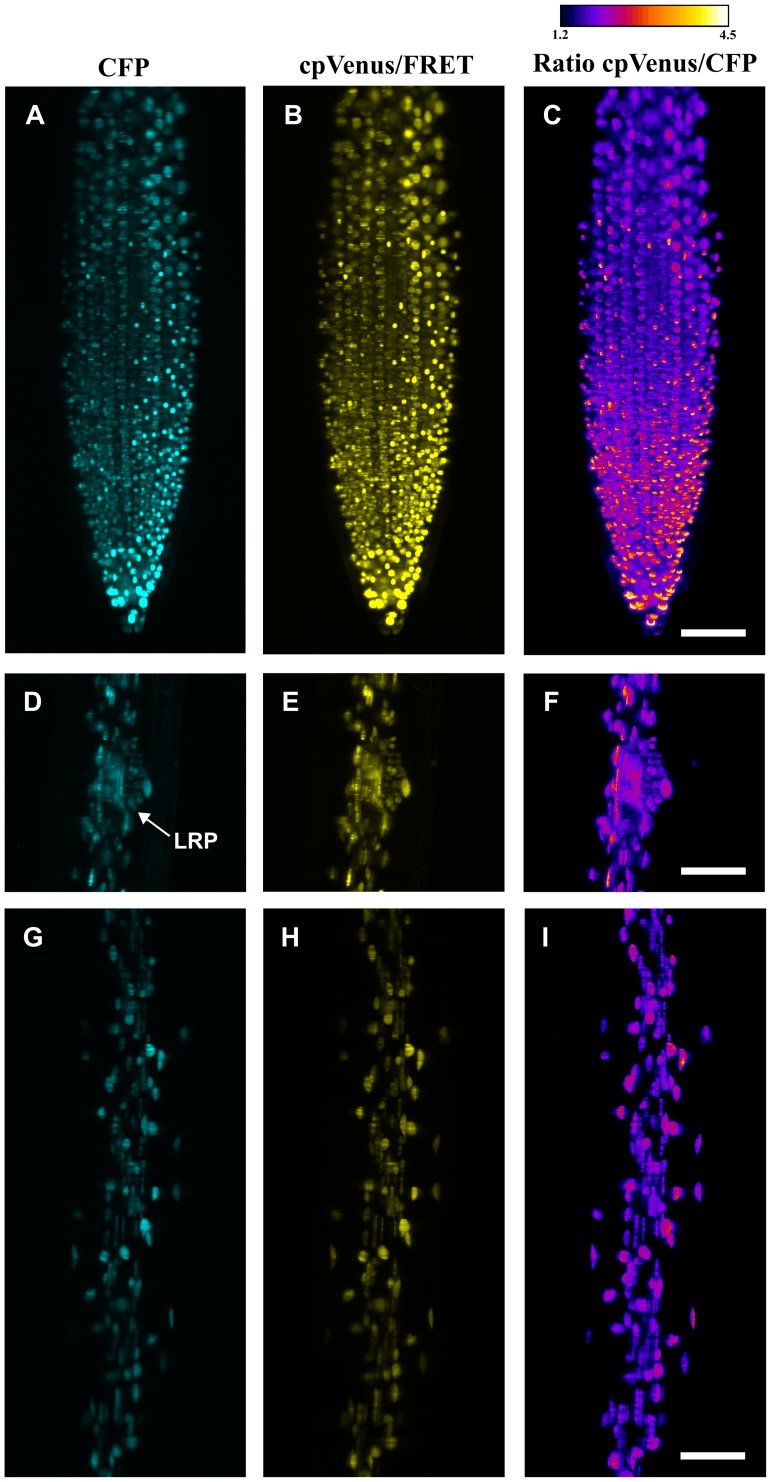
Maximum intensity projections of the stacks obtained for CFP (A,D,G), cpVenus/FRET (B,E,H) and Ratio between the two channels (C,F,I) in different regions of the *Arabidopsis thaliana* root (primary root tip, lateral root primordium and root mature zone) expressing the nuclear localized Cameleon. LRP: lateral root primordium. Scale bar is 50 µm.

Three different regions of the seedling root (primary root tip, lateral root primordium and root mature zone) are shown in Figure 2. The raw data corresponding to the primary root tip are presented in Figure S1. Single nuclei can be distinguished not only in the thicker mature zone, but also in developing organs such as the lateral root primordium, where cells are still clustered together (see also Movies S1, S2, S3). The achievable resolution depends on scattering and absorption properties of the sample and the image quality decreases as the optical path length increases. Therefore, the side of the sample which is closer to the camera tends to be sharper than the opposite side (see also Figure S1). However, notwithstanding the presence of strong scattering at the excitation wavelength (442 nm), these images show sub cellular details in depth over a large tissue volume (root diameter is approximately 100 µm). Further improvement of the image quality could be achieved using multi-view SPIM [34], multi-directional SPIM [31] or two-photon excitation [35]. It is worth noting that the use of water immersion objectives substantially improves the quality of the image compared to other approaches in which the sample is immersed in a cuvette and air objectives are used: image de-convolution [29] was not applied to the present data. The 3D reconstructions of the acquired root regions (Movies S1, S2, S3) show the SPIM ability to provide high resolution imaging over a large part of the specimen, a result that is difficult to reach with standard CLSM [28], [36].

### FRET response

One of the current and future challenges for biologists is the possibility to perform analyses of single cells laying in their natural context avoiding undesired environmental perturbation. In this view, SPIM has proved suitable for long-term visualization of Arabidopsis root growth in near physiological conditions during imaging [28], [29]. A further step is to use this microscopy modality to monitor intracellular responses, such as stimulus-induced Ca^2+^ mobilization, at single cell resolution and in unstressed conditions. In order to reach this goal we performed a series of experiments with Arabidopsis seedling expressing nuclear or cytosolic localized Cameleon YC3.6 [19].

**Figure 3:**
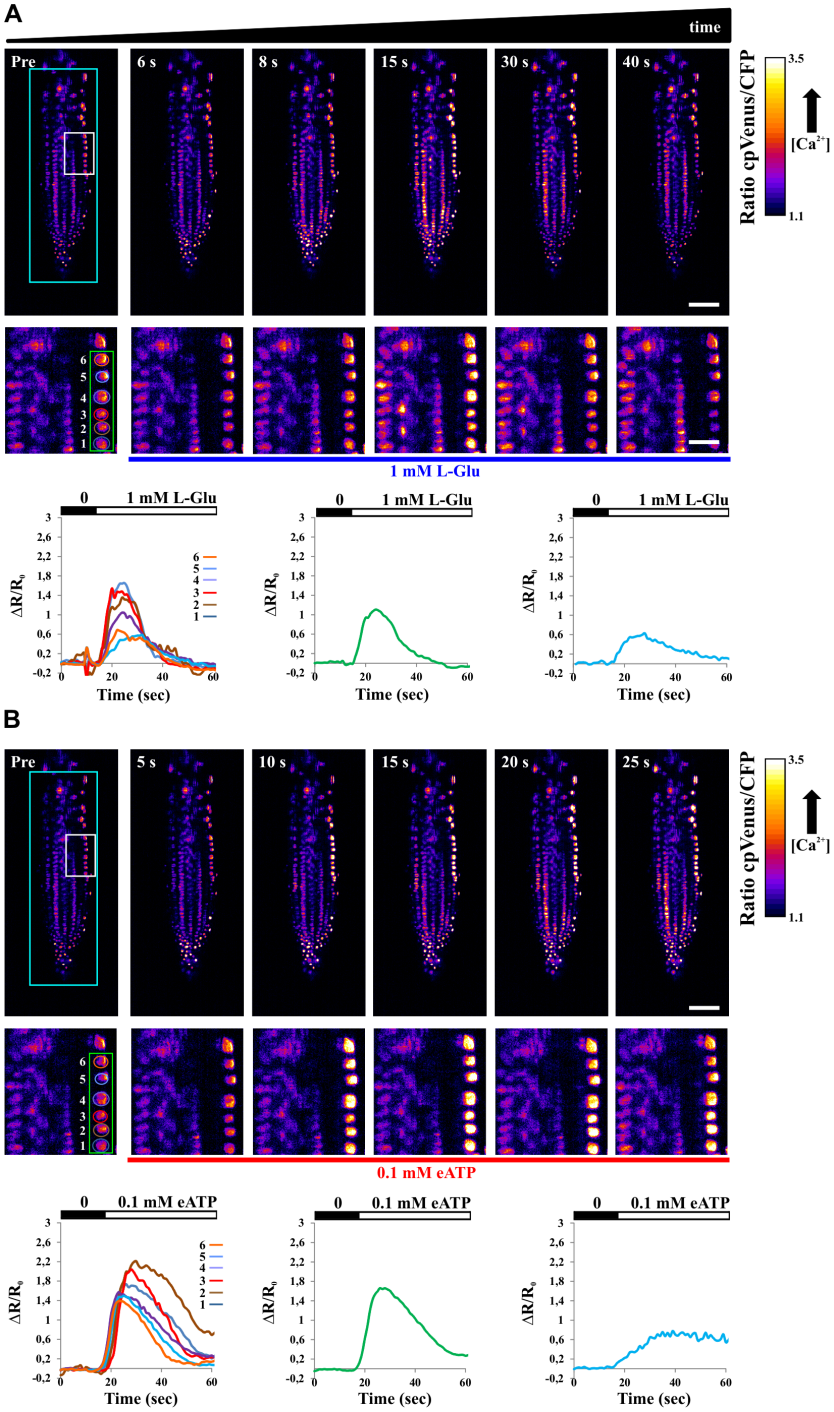
Single plane FRET ratio in root tip of Arabidopsis seedlings expressing the nuclear localized Cameleon, measured at different times in the course of 1-Glu (A) and 0.1 mM eATP (B) stimuli. (A) First row: FRET ratio images at different time points from the sensing (Pre) of the L-Glu stimulus. Second row: close up (white rectangle in the top-left image) of the FRET ratios including 6 selected nuclei (green rectangle). Third row: temporal evolution of the FRET ratio in the 6 distinct nuclei (left), average on the 6 nuclei (center), average on the entire plane (right). (B) First row: FRET ratio images at different time points from the sensing (Pre) of the eATP stimulus. Second row: close up (white rectangle in the top-left image) of the FRET ratios including 6 selected nuclei (green rectangle as in panel A). Third row: temporal evolution of the FRET ratio in the 6 distinct nuclei (left), average on the 6 nuclei (center), average on the entire plane (right). Scale bar is 50 µm and 10 µm for low and high magnifications respectively.

Figure 3 shows the FRET ratio measured in the nuclei of a single plane of the primary root of Arabidopsis expressing the nuclear localized Cameleon. Figure 3A presents the nuclear response after treatment with 1 mM Glutamate (L-Glu), a stimulus known to induce Ca^2+^ rises in plant cells [37]. Indeed, the treatment induces a Ca^2+^ peak in the cells of the entire root tip. This is visualized as a change in the FRET ratio, which corresponds to Ca^2+^ variations (the higher the ratio the higher the Ca^2+^ concentration [21]). A more detailed analysis reveals that the response to L-Glu was primarily sensed by the nuclei of the lateral root cap cells, followed by the response of the nuclei located in the upper part of the root tip (see Movie S4). Additionally distinct individual responses of single cells can be observed: the Ca^2+^ peaks measured on 6 distinct nuclei (highlighted in green in Figure 3A) show differences both in amplitudes and temporal delay (lower panel, left). Averaging the curves over the six nuclei leads to a broader time curve (Figure 3A, lower panel, center) and when the average is calculated on the entire plane we can observe a considerably longer response (Figure 3A, lower panel, right), which mimics the response achievable at lower resolution [38].

Few minutes after the recovery from the treatment with L-Glu, the same root was treated with a second stimulus consisting in external ATP (0.1 mM eATP), which has also been reported to induce intracellular Ca^2+^ rise in plant cells [19], [20], [24]. As for L-Glu, the cells of the lateral root cap primarily sensed the eATP followed by the nuclei located in the transition and elongation zones (Movie S5). It is worth noting that the images were acquired with SPIM in the same plane of the sample in the course of the two stimuli and the very same nuclei are visualized in Figure 3A, B. In particular, for the 6 nuclei (labeled in green in Figure 3A, B) the L-Glu stimulus produces single and narrow peaks, whereas eATP induces a more sustained Ca^2+^ transient, pointing out to the existence of different mechanisms responsible for the generation of the observed triggered Ca^2+^ dynamics.

Each experiment (injection of L-Glu first and eATP later) was repeated n = 6 times. The reproducibility of the responses is shown in Figure S2A, B. We observed similar responses in all experiments whereas in n = 3 control experiments (injection of the solution without any Ca^2+^ mobilizing agent) the FRET changes were below the camera noise.

**Figure 4:**
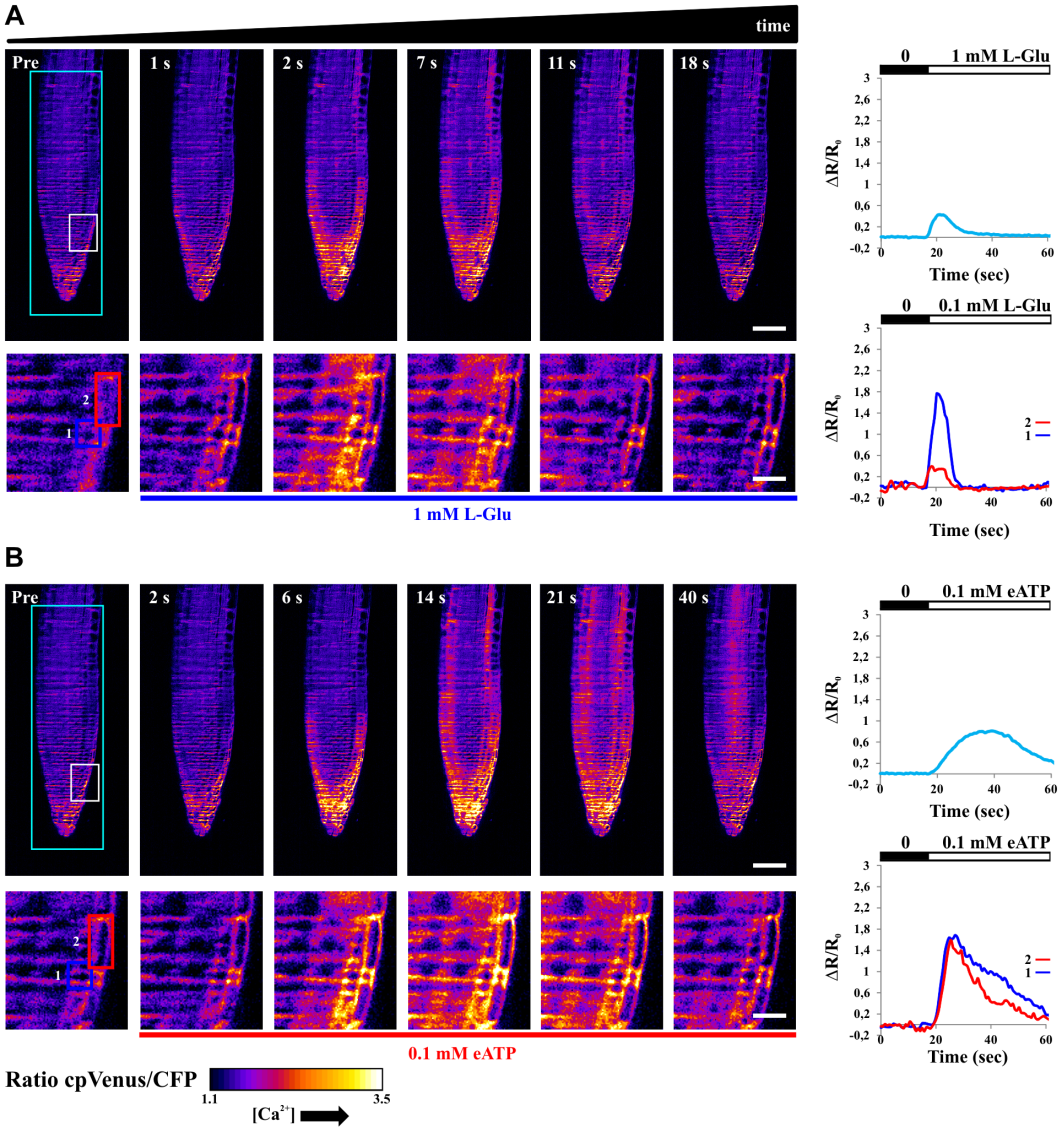
Single plane FRET ratio in root tip of Arabidopsis seedlings expressing the cytosolic localized Cameleon, measured at different times in the course of 1-Glu (A) and 0.1 mM eATP (B) stimuli. (A) Upper images: FRET ratio measured at different time points from the sensing (Pre) of the L-Glu stimulus. Lower images: close up (white rectangle in the top-let image) of the same selected FRET ratios. Upper graph: temporal evolution of the FRET ratio for the entire region highlighted by the cyan rectangle. Bottom graph: temporal evolution of the FRET ratios for two selected cells (blue and red rectangles in the lower images). (B) Upper images: FRET ratio measured at different time points from the sensing (Pre) of the eATP stimulus. Lower images: close up (white rectangle in the top-let image) of the same selected FRET ratios. Upper graph: temporal evolution of the FRET ratio for the entire region highlighted by the cyan rectangle. Bottom graph: temporal evolution of the FRET ratios for two selected cells (blue and red rectangles in the lower images). Scale bar is 50 µm and 9 µm for low and high magnifications respectively.

The comparison of the two stimuli demonstrates that SPIM offers sufficient spatial and temporal resolution to appreciate single cell responses, while monitoring large sample sizes. In Figure 3, nuclei appear as isolated and well-defined fluorescent spots, facilitating the analyses of single cells. In order to fully validate the SPIM approach, we repeated the experiments in root tip cells of Arabidopsis seedlings expressing the cytosolic localized Cameleon [19]. Figure 4A, B show the response of a root tip to L-Glu and eATP respectively, again acquired on the same plane with SPIM. In accordance to what observed with nuclei, the response to the two stimuli was primarily sensed by lateral root cap cells, followed by the response of cells present in deeper tissue, with a spread of [Ca^2+^] rises towards the cells of the transition and elongation zones (see Movie S6, S7 where Ca^2+^ propagation/percolation is observable). The comparison of the two stimuli (L-Glu and eATP), performed by plotting the average FRET responses of the entire imaged plane (cyan curve in Figure 4A, B), allows one to appreciate that L-Glu and eATP produced similar Ca^2+^ peak amplitudes with different dynamics, being eATP able to induce more sustained Ca^2+^ rises than L-Glu (see also Figure S2A, B). Double peaks are also present at longer times, as shown later. Conversely, the analysis performed in two selected lateral root cap cells (blue and red curves in Figure 4A, B) shows that they respond with very different peak amplitudes in the course of L-Glu administration. Instead, their response is similar with eATP. These data, in agreement with previous reports [38], [39], indicate that the kinetic analysis of an averaged response does not necessarily reflect the response of a single cell.

**Figure 5:**
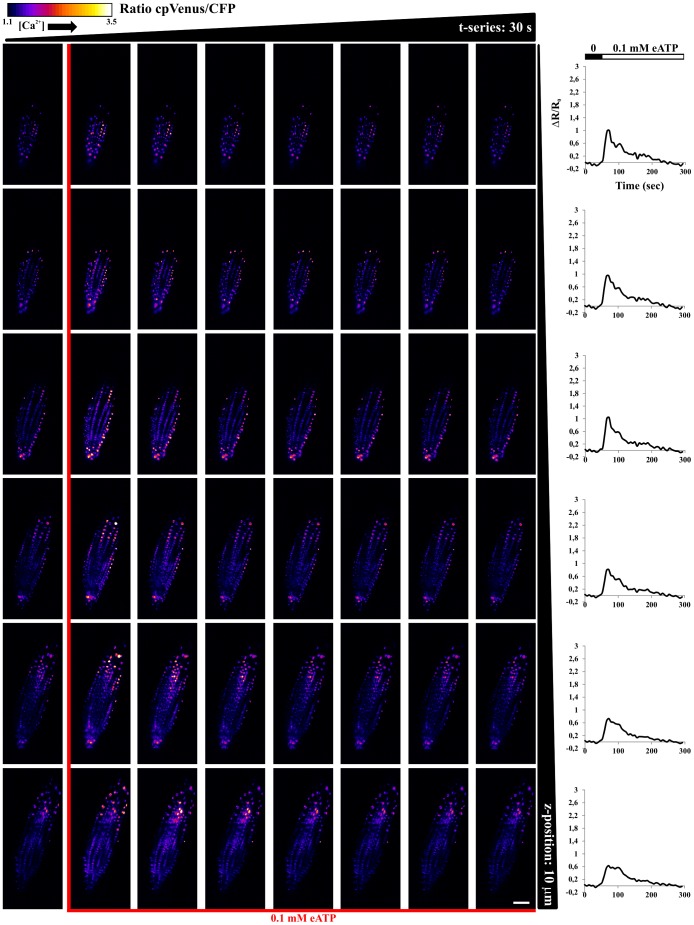
FRET ratio imaged in space and time, with 10 µm steps (columns) and every 30 s (rows), in root tip of Arabidopsis seedlings expressing the nuclear localized Cameleon, in the course of 0.1 mM eATP stimulus. Graphs on the right hand side show temporal evolution of the averaged FRET ratio for each single selected plane. Scale bar is 50 µm.

**Figure 6:**
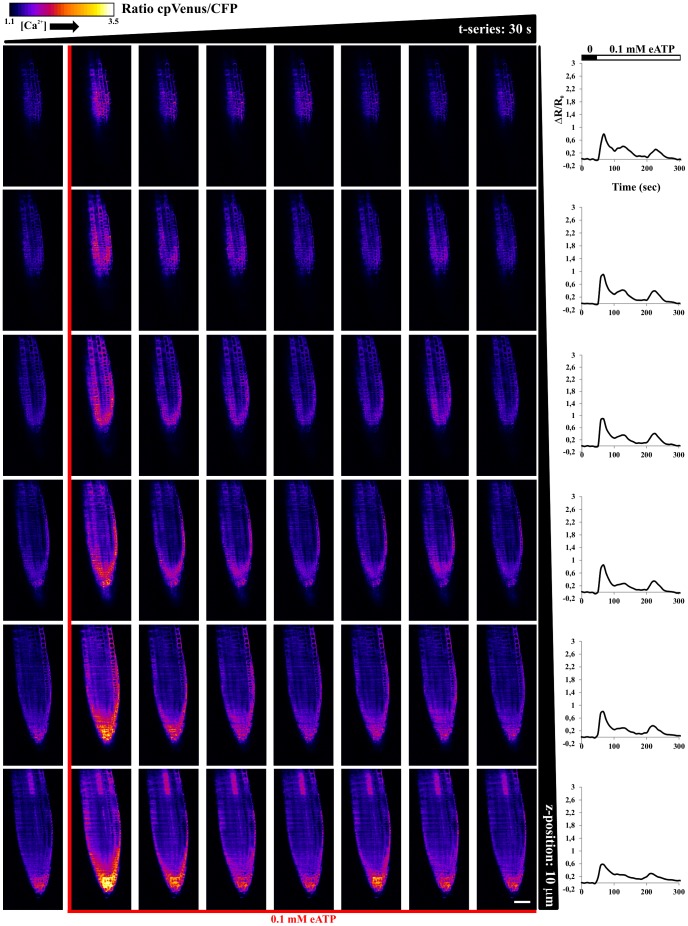
FRET ratio imaged in space and time, with 10 µm steps (columns) and every 30 s (rows), in root tip of Arabidopsis seedlings expressing the cytosolic localized Cameleon, in the course of 0.1 mM eATP stimulus. Graphs on the right hand side report temporal evolution of the average FRET ratio for each single selected plane. Scale bar is 50 µm.

Although understanding the mechanisms underlying the observed differences in terms of nuclear and cytosolic Ca^2+^ dynamics in response to the two stimuli, is beyond the aim of the present work, the different responses demonstrate that the use of SPIM with plants expressing a genetically encoded Ca^2+^ probe, offers an adequate resolution to perform single cell analysis over a complex organ. Moreover, the fast acquisition rate (typically 2–10 Hz for a single plane), enables one to obtain an easy visualization of Ca^2+^ signal percolation in plants (see also Movies S4, S5, S6, S7) [39]. In order to demonstrate the SPIM ability to perform 3D analysis we treated primary root tips of Arabidopsis seedlings with 0.1 mM eATP, acquiring adjacent image planes (typically 10 planes, with 5–10 µm steps, every 5–8 s), in the course of the stimulus. Figure 5 and 6 show the FRET ratio imaged at different times and depths (Z-position). Note that only a subset of the acquired planes and time points are presented. The temporal FRET responses, averaged on each plane, allows one to observe that the entire organ responds to eATP, with differences among planes. In particular, every plane shows different responses in terms of peak amplitudes and dynamics (see graphs in the right hand side of Figure 5, 6). The cytosolic Ca^2+^ dynamics present several oscillations, whereas the responses of the nuclei show less resolved peaks, as we previously observed with wide-field microscopy [20]. A 3D reconstruction of the sample over time is possible, as shown in Movies S7, S8. In this case, the entire root tip can be visualized, allowing one to observe fine details of the nuclei and the cytosols. At the same time, it is possible to observe the systemic root tip response. Similar results could not be reached by means of wide-field microscopy (Figure S3A), which can be used to visualize large plant volumes, due to the lack of optical sectioning and single cell resolution.

### Comparison with CLSM

Single cell visualization of intracellular Ca^2+^ dynamics in a root tip layer has been reported by some authors [24], [40] along with a recently published detailed protocol to image Cameleon with CLSM has been recently published [41]. The results presented in Figure 3, 4 show that SPIM is indeed an alternative approach to CLSM, in terms of imaging quality. SPIM has lower spatial resolution than CLSM (Figure S3), but it is still able to offer subcellular detail in a large tissue volume. SPIM seems to have a higher temporal resolution than standard CLSM since it allows one to study Ca^2+^ dynamics in multiple planes in a time window compatible with relatively fast (few seconds) biological events. In particular, when 3D reconstructions are taken into consideration the photobleaching caused by the selective illumination of single planes has been reported to be orders of magnitude lower than the one caused by CLSM [27], [42]. A further advantage is the simplicity of the setup and its low cost (for an estimate of the cost see Ref. [30].

Although a quantitative comparison of the techniques strongly depends on the actual CLSM microscope employed, a parallel series of experiments were carried out with CLSM and SPIM (Figure S3B, C). The experiments consisted in the injection of 0.1 mM of eATP to induce an increase and oscillation in Ca^2+^, lasting up to 5 min (Figure S3A). In SPIM, some horizontal lines appeared in the images, caused by the presence of shadows within the light sheet. These lines can however be removed by hardware [31]. The contrast is slightly lower with SPIM, mainly because of the noise of the camera (note that in Figure S3A background subtraction was not applied). Nevertheless, we observed a stronger response with SPIM (+35±10%, n = 3), thanks to the higher dynamic range of the camera. Finally a larger region of the sample could be imaged with SPIM.

Hence, SPIM combines the advantages of different microscopy approaches in a single technique. As a matter of fact, single cell resolution, typical of CLSM techniques is coupled with the ability to observe systemic root responses, characteristic of wide field microscopy.

## Conclusions

The above presented data indicate that the developed SPIM-FRET method is ideally suited to monitor Ca^2+^ signaling *in vivo*, at high resolution, with negligible photo-bleaching and in depth over a large tissue volume. The instrument is relatively simple and cost effective. Such technology could lead to many different experiments that combine developmental programs with environmental stimuli. Imaging of Ca^2+^ using SPIM-FRET is not limited to plant biology but can be used for 3D mapping of Ca^2+^ dynamics in a variety of semi-transparent biological samples including embryos such as zebrafish (*Danio rerio*) and *Drosophila melanogaster*.

## Supporting Information

Figure S1 Sagittal (A–C) and two transverse sections (D–I) of the specimen (Arabidopsis expressing the nuclear localized Cameleon) for CFP signal, cpVenus and FRET ratio. The sagittal sections are acquired within approximately one third of the sample thickness. The transverse sections are obtained scanning the entire sample within the light sheet. Scale bar is 50 µm.
(TIF)

Figure S2 Statistical analysis of the response amplitude and duration, in root tip of Arabidopsis seedlings expressing the nuclear or cytosolic localized Cameleon, for 0.1 mM eATP and 1 mM L-Glu stimuli. (A) Mean value of the FRET ratio changes measured on the peak of the response (ΔR_max_/R_0_). (B) Mean value of the FRET ratio changes measured 40 s after the stimulus (ΔR_t40_/R_0_). The eATP response is still active at long times while the L-Glu response is depleted. Values are means ± SE (n = 6). p-values were calculated by Student's t test.
(TIF)

Figure S3 Comparison of different microscopy modalities for measuring eATP-induced Ca^2+^ dynamics in root tip of Arabidopsis seedlings expressing the cytosolic localized Cameleon. Selected FRET ratios images of the root tip at different time points from the sensing (Pre) of the eATP stimulus acquired with: (A) Wide-field microscopy with a 20× detection objective as described in Ref. [20]; (B) CLSM analysis. The images were acquired with a 63× water immersion objective as described in Ref. [41]; (C) SPIM microscopy. The images were acquired with a 20× objective as described in Material and methods. Scale bar is 50 µm. Background subtraction was not applied to these experiments.
(TIF)

Movie S1 Three-dimensional (3D) reconstruction of the primary root tip of *Arabidopsis thaliana* (CFP fluorescence signal) expressing the nuclear localized Cameleon.
(MP4)

Movie S2 Three-dimensional (3D) reconstruction of a lateral root primordium of *Arabidopsis thaliana* (CFP fluorescence signal) expressing the nuclear localized Cameleon.
(MP4)

Movie S3 Three-dimensional (3D) reconstruction of the root mature zone of *Arabidopsis thaliana* (CFP fluorescence signal) expressing the nuclear localized Cameleon.
(MP4)

Movie S4 Time series of nuclear FRET ratio images of an Arabidopsis seedling root tip expressing the nuclear localized Cameleon challenged with 1 mM L-Glu. The movie plays 4 times at real-time.
(AVI)

Movie S5 Time series of nuclear FRET ratio images of an Arabidopsis seedling root tip expressing the nuclear localized Cameleon challenged with 0.1 mM eATP. The movie plays 4 times at real-time.
(AVI)

Movie S6 Time series of cytosolic FRET ratio images of an Arabidopsis seedling root tip expressing the cytosolic localized Cameleon challenged with 1 mM L-Glu. The movie plays 4 times at real-time.
(AVI)

Movie S7 Time series of cytosolic FRET ratio images of an Arabidopsis seedling root tip expressing the cytosolic localized Cameleon challenged with 0.1 mM eATP. The movie plays 4 times at real-time.
(AVI)

Movie S8 Time lapse 3D reconstruction of nuclear FRET ratio of an Arabidopsis seedling root tip expressing the nuclear localized Cameleon challenged with 0.1 mM eATP. The movie plays 20 times at real-time.
(AVI)

Movie S9 Time lapse 3D reconstruction of cytosolic FRET ratio of an Arabidopsis seedling root tip expressing the cytosolic localized Cameleon challenged with 0.1 mM eATP. The movie plays 20 times at real-time.
(AVI)
