# Supplementary figures and images for: Calcium Dynamics in Root Cells of Arabidopsis thaliana Visualized with Selective Plane Illumination Microscopy
Source: PLoS One. 2013 Oct 16;8(10):e75646. doi: 10.1371/journal.pone.0075646 (PMC3797704; doi:10.1371/journal.pone.0075646)

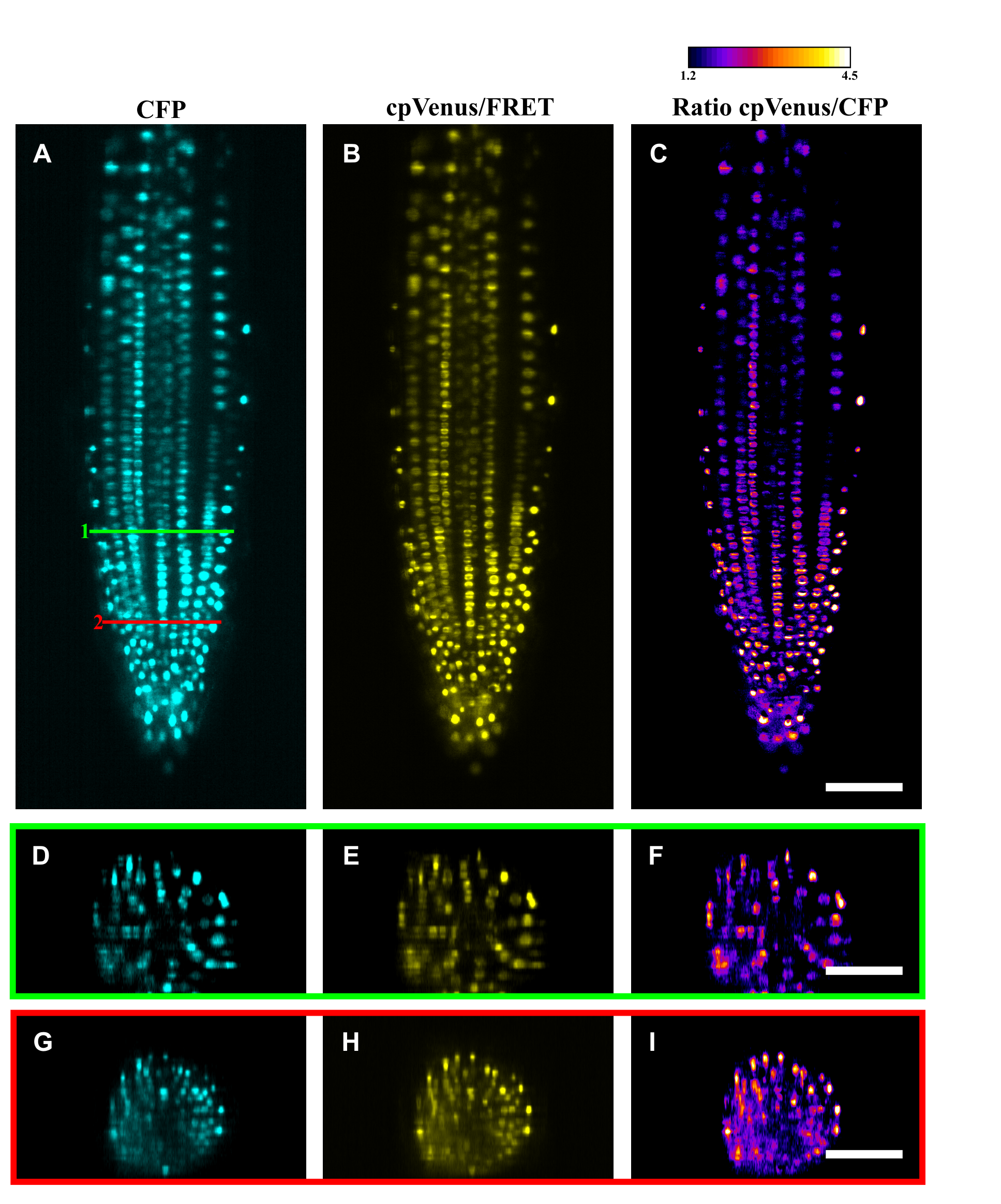

Supplement: Figure S1 — Sagittal (A–C) and two transverse sections (D–I) of the specimen (Arabidopsis expressing the nuclear localized Cameleon) for CFP signal, cpVenus and FRET ratio. The sagittal sections are acquired within approximately one third of the sample thickness. The transverse sections are obtained scanning the entire sample within the light sheet. Scale bar is 50 µm. (TIF) [file pone.0075646.s001.tif]

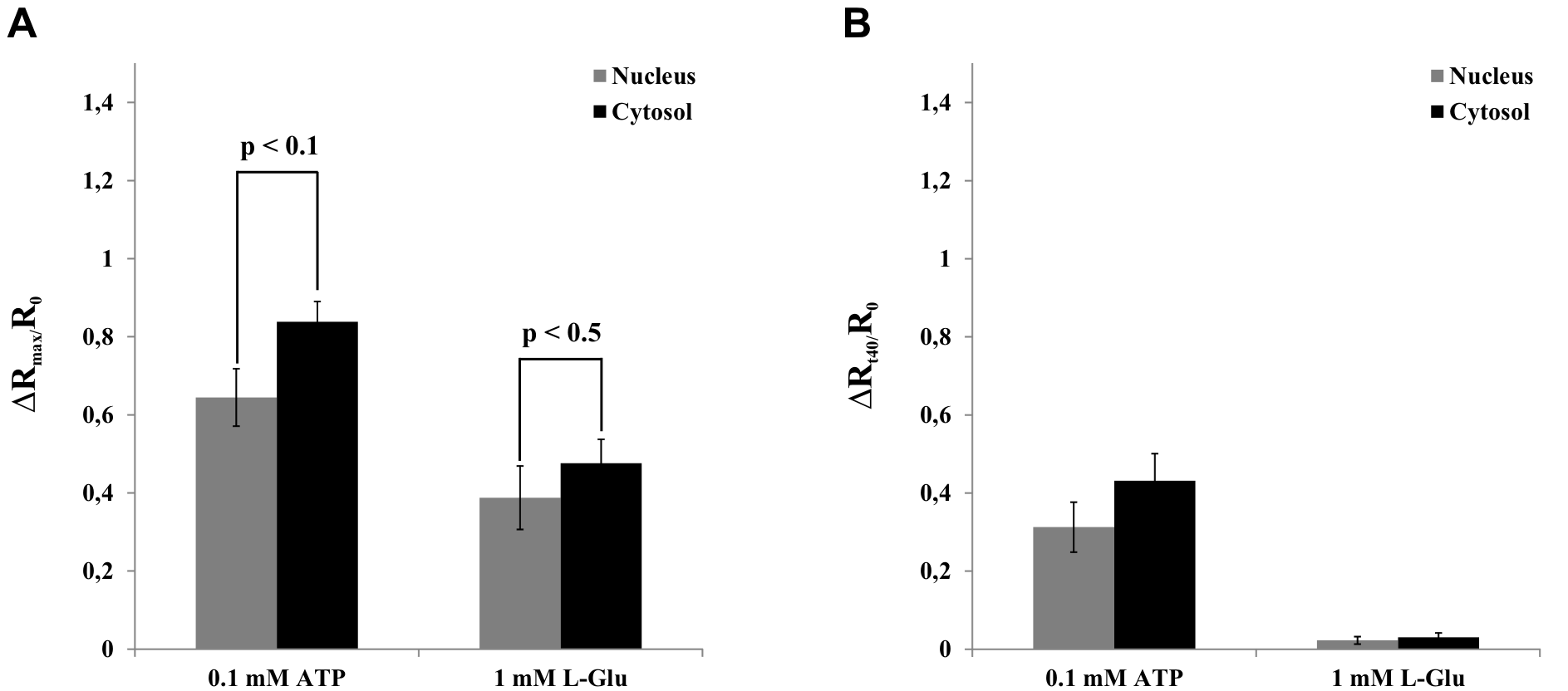

Supplement: Figure S2 — Statistical analysis of the response amplitude and duration, in root tip of Arabidopsis seedlings expressing the nuclear or cytosolic localized Cameleon, for 0.1 mM eATP and 1 mM L-Glu stimuli. (A) Mean value of the FRET ratio changes measured on the peak of the response (ΔRmax/R0). (B) Mean value of the FRET ratio changes measured 40 s after the stimulus (ΔRt40/R0). The eATP response is still active at long times while the L-Glu response is depleted. Values are means ± SE (n = 6). p-values were calculated by Student's t test. (TIF) [file pone.0075646.s002.tif]

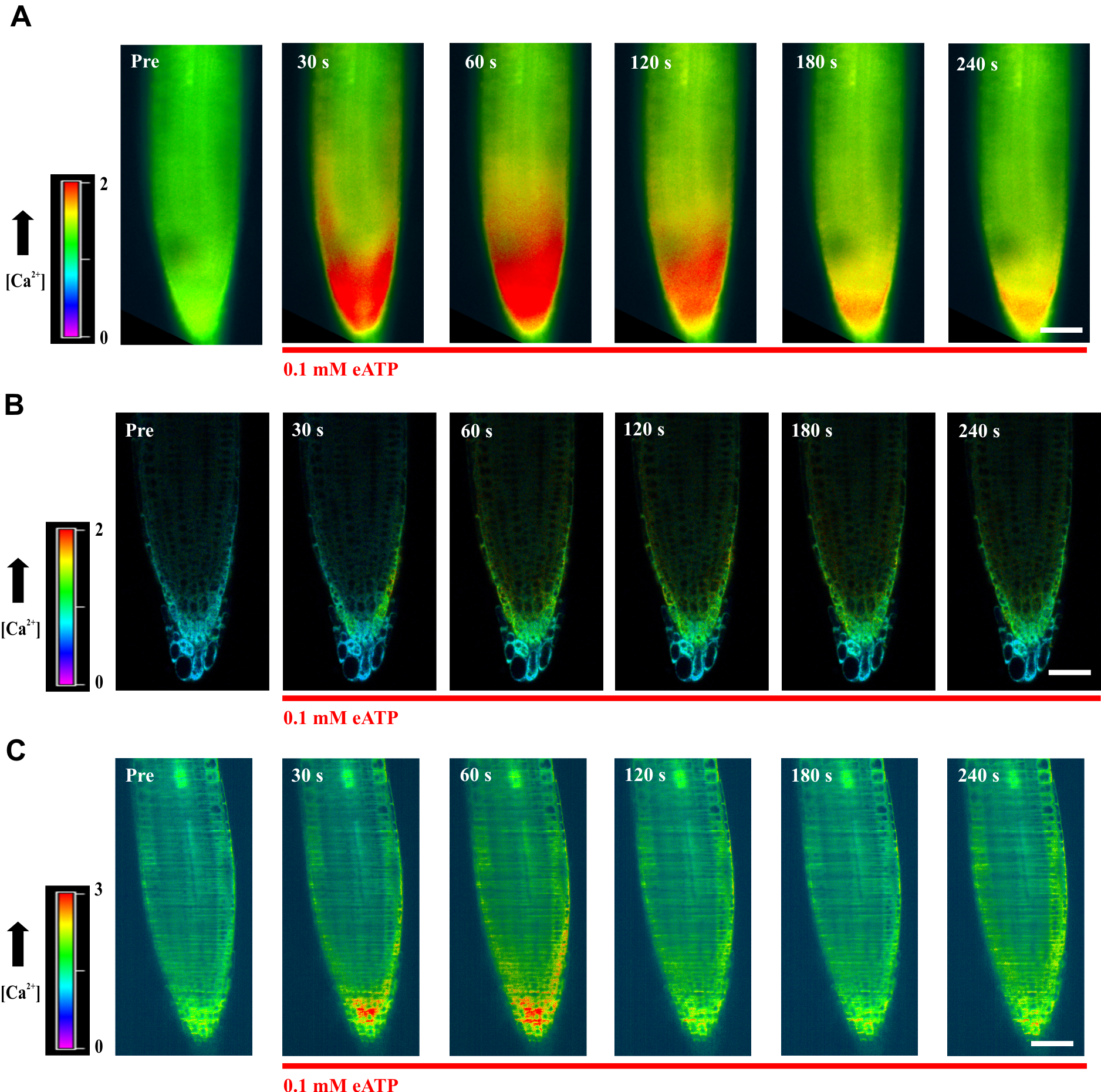

Supplement: Figure S3 — Comparison of different microscopy modalities for measuring eATP-induced Ca2+ dynamics in root tip of Arabidopsis seedlings expressing the cytosolic localized Cameleon. Selected FRET ratios images of the root tip at different time points from the sensing (Pre) of the eATP stimulus acquired with: (A) Wide-field microscopy with a 20× detection objective as described in Ref. [20]; (B) CLSM analysis. The images were acquired with a 63× water immersion objective as described in Ref. [41]; (C) SPIM microscopy. The images were acquired with a 20× objective as described in Material and methods. Scale bar is 50 µm. Background subtraction was not applied to these experiments. (TIF) [file pone.0075646.s003.tif]
